# Effect of fermentation, malting and ultrasonication on sorghum, mopane worm and *Moringa oleifera*: improvement in their nutritional, techno-functional and health promoting properties

**DOI:** 10.3389/fnut.2024.1469960

**Published:** 2024-10-02

**Authors:** Mpho Sebabiki Maleke, Oluwafemi Ayodeji Adebo, Jonathan Wilkin, Moira Ledbetter, Xi Feng, John Gieng, Tumisi Beiri Jeremiah Molelekoa

**Affiliations:** ^1^Centre for Innovative Food Research (CIFR), Department of Biotechnology and Food Technology, Faculty of Science, University of Johannesburg, Doornfontein, Johannesburg, South Africa; ^2^Division of Engineering and Food Science, School of Applied Sciences, Abertay University, Dundee, United Kingdom; ^3^Department of Nutrition, Food Science, and Packaging, San Jose State University, San Jose, CA, United States

**Keywords:** traditional processing, novel processing, nutritional benefits, underutilised, edible insect

## Abstract

**Background:**

Food processing offers various benefits that contribute to food nutrition, food security and convenience. This study investigated the effect of three different processes (fermentation, malting and ultrasonication) on the nutritional, techno-functional and health-promoting properties of sorghum, mopane worm and *Moringa oleifera.*

**Methods:**

The fermented and malted flours were prepared at 35°C for 48 h, and for ultrasonication, samples were subjected to 10 min at 4°C with amplitudes of 40–70 Hz. The biochemical, nutritional quality and techno-functional properties of the obtained flours were analysed using standard procedures.

**Results:**

Fermentation resulted in significantly lower pH and higher titratable acidity in sorghum and mopane worm (4.32 and 4.76; 0.24 and 0.69% lactic acid, respectively), and malting resulted in higher total phenolic content and total flavonoid content in sorghum (3.23 mg GAE/g and 3.05 mg QE/g). Ultrasonication resulted in higher protein and fibre in raw sorghum flour (13.38 and 4.53%) and mopane worm (56.24 and 11.74%) while raw moringa had the highest protein (30.68%). Biomodification by fermentation in sorghum led to higher water and oil holding capacity and increased dispersibility in the ultrasonicated samples. Ultrasonication of mopane worms led to higher water holding capacity, oil holding capacity and dispersibility. Lightness was found to be significantly higher in the fermented samples in sorghum and mopane worm. Raw moringa had the greatest lightness compared to the ultrasonicated moringa. Moringa had the most redness and browning index among all samples.

**Conclusion:**

In this study, all the investigated processes were found to have caused variations in flours’ biochemical, nutritional and techno-functional properties. Ultrasonication process was noteworthy to be the most efficient to preserve the nutritional value in sorghum, mopane worm and *M. oleifera* flours.

## 1 Introduction

The world population has been reported to reach above 7.9 billion since 2022, and it is being predicted to slowly increase as the population increases (1). The population growth rate has been a concern due to its implications on resources, the environment, and social and economic systems. In recent decades, there has been an increasing awareness of the significance of a nutritious diet, prompted by concerns about the increase in obesity, diabetes, cardiovascular diseases, and other diet-related health issues (2). Several dietary trends have been reported (3, 4) from the adaptation of wholly plant-based diets to the inclusion of edible insects or flexitarian diets emphasising the consumption of fruits, vegetables, whole grains, and legumes. However, health issues, environmental concerns, and ethical considerations are the driving forces for this change (3).

Concerns about environmental sustainability have influenced the eating habits of individuals, hence there is a growing interest in consuming locally sourced and sustainably produced foods (2). Another challenging issue is malnutrition which remains a pressing issue in many nations, posing a significant risk factor for disease burden and mortality rates (1). The estimated 2 billion individuals that are affected by the detrimental effects of malnutrition extend far beyond physical health, impacting cognitive development and social skills (4, 5). Children who experience prolonged malnutrition may suffer from delayed physical growth and motor development, reduced intellectual quotient (IQ) and increased behavioural problems (5). Furthermore, the insufficient intake of essential nutrients such as proteins, vitamins, and minerals present a significant challenge for communities in developing countries, where undernutrition and related disorders are prevalent, which further highlights the necessity of consuming local indigenous crops and related products (4).

Sorghum is an indigenous crop consumed across Africa and India and has gained more consumer interests being a gluten-free alternative, high bioactive compounds, and fibre content (5). It is considered a valuable crop that can withstand drought-prone areas of Africa and India. Researchers have used sorghum as a whole grain or an additive to increase its value. Sorghum is known for its high phytochemical content, which contains phenols, tannins, anthocyanins, hydroxybenzoic acids, hydroxycinnamic acids, and other flavonoids (6). These phenolic compounds have a substantial influence on human health and provide antioxidant action (6). However, its protein and nutritional quality restrict its use as human food. Therefore, there is a need to include high-protein foods to sorghum-based foods to improve the protein content and digestibility. There is a variety of high-protein foods such as legume flour and whey protein. Recently, interests have moved away from more conventional food to edible insects such as cricket and mopane worms (4). These insects are high in protein and considered underutilised; despite their nutritional benefits they have not been significantly used in food products (7). Mopane worms (*Gonimbrasia belina*), are a unique and fascinating culinary delicacy deeply rooted in the traditional diets of certain African communities (8). The mopane worms have been a traditional staple food mostly in the Limpopo province in South Africa, Botswana and Zimbabwe. They have been reported to be a valuable food source due to their nutritional composition, especially in areas where conventional cattle production may be difficult (8). They make a substantial contribution to the nutritional needs by offering a well-balanced blend of protein (60–70%), lipids (15–20%), vitamins, and minerals (7).

Moringa plant (*Moringa oleifera*) is prioritised for its medicinal properties and several other uses, with the majority of its components are edible. Its defined seeds, blooms, and fruits (pods) provide pertinent nutrients and chemicals for feeding as characterised by (9). Moringa is an important plant in India, the Philippines, Ethiopia as well as Sudan though it popularly grown in the South, East and West Africa. *M. oleifera* leaf powder (MOLP) generated from the tree leaves; is widely acclaimed for its outstanding nutritional profile (10). A few studies have taken a methodical approach to the supplementation of food with MOLP, highlighting the usage of *M. oleifera* as a natural additive and as a functional food (11–13). The MOLP has been reported to have shown all the nine essential amino acids, hence it is considered a complete protein source (10). Hence blending MOLP with sorghum and mopane worm will be able to develop a food product that is not only nutritionally better but also environmentally sustainable and culturally suitable while also addressing various areas of food security and health.

Food processing plays a vital role in ensuring food safety, accessibility, and convenience while extending shelf life and bridging the gap between supply and demand (14). It can enhance the nutritional value, safety, and quality of food, despite concerns about additives in processed foods. Traditional food processing methods involve time-tested techniques like drying, fermenting, and salting, while novel food processing leverages modern technologies such as high-pressure processing, pulsed electric fields, ultrasonication and nanotechnology to enhance food quality, safety, and shelf life (14). The nutritional and health profile of sorghum, insects and moringa has been investigated mostly using fermentation and malting (15, 16). However, it appears that these approaches did not fully explore the comparison of the aforementioned processes and ultrasonication. Ultrasonication has been reported to activate enzymes naturally present in food by increasing enzymatic activity, which can have a positive effect depending on the specific enzymes involved (17). Ultrasonication activates naturally occurring enzymes in food by creating conditions that facilitate enzymatic reactions via cavitation, microstreaming, mechanical effects, thermal effects, and sonochemistry (18). For instance, it can activate enzymes responsible for contributing to nutritional quality or initiate enzymatic reactions that can lead to undesirable changes in food quality (17). It can also be used as an additional step to help reduce microbial load, therefore increasing the shelf life (18). The exact management of ultrasonication settings is required to obtain the optimum amount of enzyme activation while avoiding adverse effects.

In a study by Awobusuyi et al. (15), sorghum was blended with edible insect and in Mridula et al. (16) it was blended with sunflower and peanut flour to produce cookies with improved protein. Mridula et al. (18) found that cookies with a sorghum substitution level of up to 50% could be made using composite flours made of wheat and sorghum. Sorghum can be used to replace some of the wheat flour in bread, cookies, and other snacks, according to some reported studies (19, 20). However, studies are limited on the use of sorghum and mopane worm blends into food products. As a result, this present study explored the effect of three different processes (fermentation, malting and ultrasonication) on the nutritional, functional and health-promoting properties of sorghum, mopane worm and *M. oleifera* to develop a health-promoting and value-added snack.

## 2 Materials and methods

### 2.1 Material

White raw sorghum grains (SG) were purchased from AGT (Krugersdorp, South Africa) and mopane worms (MW) were bought from a local market (Johannesburg, South Africa) in dry form and packaged in plastic containers. *M. oleifera* (MO) leave powder was procured from a health store (Dischem, Johannesburg, South Africa). All analytical-grade chemicals and reagents that were used during the experiment were purchased from Merck (Darmstadt, Germany) and other reputable suppliers.

### 2.2 Methods

#### 2.2.1 Flour sample preparation of SG and MW

The SG and MW were removed from foreign components (stones, stalks), washed, and kept in airtight containers at room temperature until further usage. A portion (200 g) of cleaned SG (another portion reserved for malting) and MW were milled using Philips Mill HR2056/90 (Koninklijke Philips N.V., Eindhoven, Netherlands) into flour for fermentation and ultrasonication process. The milled SG and MW were then labelled as raw sorghum flour (RSF) and raw mopane worm (RMW), respectively. The moringa was purchased already in powder form (RMO) and was sieved to obtain a fine powder and the resulting flour was stored at 4°C in Ziploc bags for further analysis.

#### 2.2.2 Processing of samples

##### 2.2.2.1 Fermentation

A portion of the obtained flours of RSF and RMW was sieved through a 500 μm sieve (Analysette 3 Spartan, Fritsch GmbH, Idar-Oberstein, Germany). The processing method followed was the probiotic fermentation using freeze-dried culture (Lactic acid bacteria, CHN-22; Hansen Holding A/S, Horsholm, Denmark). The prepared RSF and RMW flour were fermented according to the procedure previously described by Kewuyemi et al. (21) to obtain a fermented sourdough. Triplicate fermentation was performed by combining 0.4 g of starter culture with 100 g flour and 200 mL of distilled water. The produced dough was fermented for 48 h in an incubator at 35°C. The sourdoughs were subsequently subjected to freezing at −20°C followed by freeze-drying (Telstar LyoQuest freeze dryer, Terrassa, Spain). The obtained samples were kept at 4°C in Ziploc bags for further analysis.

##### 2.2.2.2 Malting

The malting process for RSF followed the method as outlined by Ojha. (22) with slight modifications Approximately 400 g of sorghum grains was washed with sterile distilled water and drained. The RSF was immersed in sterilised water at a ratio of 1:3 (w/v) at a temperature of 35°C for 48 h. The softened grains were then thoroughly washed, distributed evenly on a germination tray, covered in muslin fabric cloth and incubated at 27°C for 48 h. The grains were regularly moistened to ensure adequate hydration. Sprouted grains were later dried in a laboratory oven preheated at 50°C for 24 h. The dried grains were then milled using a laboratory miller (Platinum stand dry miller, KJ-1250, Castelfranco Veneto, Italy) and the resulting flour was sifted to obtain fine material using a sieve (500 μm) to produce a malted sorghum flour (MSF). The flour was kept in Ziploc bags at 4°C until analysis.

##### 2.2.2.3 Ultrasonication

The ultrasonication of the samples was carried out using Misonix Ultrasonic Liquid Processor (FB705, Fisher Scientific) following a procedure previously described by Lohani & Muthukumarappan; Kingwascharapong (23, 24), with minor modifications. In 100 mL of distilled water, 30 g of the samples was reconstituted in a glass beaker. Samples were then subjected to 10 min of ultrasonication at 4°C with amplitudes of 70 Hz. The samples were ultrasonicated while in a water bath with ice (to prevent heat accumulation) until they reached 37°C. The obtained samples were transferred into 50 mL centrifuge tubes and closed tightly with the lid, frozen and freeze-dried. The samples were then stored in freezer bags for further analysis.

#### 2.2.3 Analytical methods

##### 2.2.3.1 Proximate composition

The proximate components, including crude protein, crude fat, ash content, and moisture, were determined using methods described by AOAC (25). Total carbohydrate was measured by difference, whereas total energy was computed using the Atwater factors.

##### 2.2.3.2 pH and titratable acidity

The pH value was measured using a pH metre (HANNA, Woonsocket, United States) by immersing the pH probe into 10 mL of the sample. The reading was recorded as displayed on the screen (26). The amount of TTA was determined by titration of the supernatant with sodium hydroxide solution (NaOH, 0.1 N) to pH 8.3 (27). The TTA of the samples was expressed as % lactic acid according to Equations 1 and 2 below:


(1) $$\%\text{lactic}\;\text{acid}=\frac{N\;x\;V\;x\;\text{ME}\;\text{of}\;\text{lactic}\;\text{acid}\;x\;100}{\text{Weight}\;\text{of}\;\text{sample}\;\left(\text{mL}\right)}$$

Where:

N is the normality of the sodium hydroxide.

V is the volume of sodium hydroxide (mL) used to reach the titration end-point


(2) $$\begin{array}{l} \text{ME}\;\left(\text{mili}-\text{equivalent}\;\text{of}\;\text{lactic}\;\text{acid}\right)=\\ \;\frac{\text{Molecular}\;\text{weight}\;\text{of}\;\text{lactic}\;\text{acid}}{1000} \end{array}$$

##### 2.2.3.3 Extraction of samples

The processed samples were extracted following the procedure described by Arouna (28) with some minor adjustments. Approximately,0.25 g of the sample was dissolved in 5 mL of 70% aqueous methanol in a centrifuge tube. The resulting solution was sonicated (Scientech 704, Labotech, Johannesburg, South Africa) for 30 min and centrifuged (Eppendorf 5702R; Merck) for 10 min at 2500 rpm, 4°C. The recovered supernatants were placed into Eppendorf tubes and kept at a temperature of 4°C for further analysis.

##### 2.2.3.4 Total phenolic content

According to Moyo et al. (29), the Folin–Ciocalteu method was used to determine the TPC of the samples. In a 96-well microplate, 10 μL of extract was taken in triplicates, followed by 50 μL of Folin–Ciocalteu reagent. After 3 min in the dark, approximately 50 μL of 7.5% Na_2_CO_3_ was added. The plate was then covered with aluminium foil, and the absorbance was measured at 750 nm using a microplate reader (iMark, Biorad, South Africa). The results were expressed as mg gallic acid equivalents (GAE)/g, with gallic acid as standard.

##### 2.2.3.5 Total flavonoid content

To evaluate total flavonoid content, 10 μL of the extract was pipetted onto a microplate with 30 μL of 2.5% NaNO_2_ and let to stand for 5 min, as per the method by Moyo et al. (29). The next step was to add 30 μL of 1.25% AlCl_3_ and 100 μL of 2% NaOH. A microplate reader was used to test the sample’s absorbance at 450 nm. The data were presented as mg quercetin equivalents (QE)/g, with quercetin as the standard.

##### 2.2.3.6 ABTS [2,2-Azinobis (3-ethyl-Benzothiazone-6-sulfonic acid)]

The assay was performed based on the method by Kewuyemi et al. (21). The radical scavenging capacity was measured by using ABTS+ radical cation. Following the addition of 180 μL of ABTS solution and 5 min of incubation in the dark, 20 μL extract was pipetted onto a microliter plate. The solution’s absorbance was determined using a microplate reader (BioTek, Agilent Technologies, South Africa) set to 750 nm. The findings were presented as μM Trolox equivalents (TE)/g sample using Trolox as the reference solution.

##### 2.2.3.7 Ferric ion reducing antioxidant power

The FRAP test followed the procedure reported by Kewuyemi and Adebo (30). The solutions required for the experiment contained acetate buffer (300 mM, pH 3.6), diluted HCl (40 mM), 2,4,6-Tripyridyl-S-triazine (TPTZ, 10 mM), and freshly made ferric chloride hexahydrate (FeCl_3_·6H_2_O, 20 mM). Pipette 240 μL of FRAP working solution (at 37°C) into each microplate well, then add 10 μL of Trolox solution (standard solution 0 to 1 mM). In another set of wells, 10 μL of methanolic extracts were pipetted, while the control contained 75% ethanol. The plate was then incubated at 37°C for 30 min (Model: 222/227, Scientific Manufacturing CC, Cape Town, South Africa), and the resulting reaction mixture was read at 593 nm on a microplate smartReader (Accuris Instruments, United Sttaes). The FRAP of the extracts was presented as a millimolar of Trolox equivalent per gram (mM TE/g).

##### 2.2.3.8 Oil holding capacity

The oil holding capacity (OHC) and water holding capacity (WHC) of flours are important properties for in-process handling and as a result, impact customer preference for the finished product. For example, a high WHC is ideal for improved thickening capacity, hydration, and satiety, but a moderate degree of OHC is optimal for in-process fat holding, taste retention, and palatability (31). The sample’s absorption of oil was examined based on the method described by Ohizua et al. (32), with minor adjustments. In a clean 15 mL centrifugal tube, 1 g of sample flour was combined with 10 mL oil and shaken to make a homogenous mixture. The tube was then centrifuged for 20 min at 1100 × *g* after standing at ±23°C for an hour. The amount of free oil was used to calculate the oil absorption capacity percentage.

##### 2.2.3.9 Water holding capacity

The water holding capacity was determined based on the procedure (33). About 0.5 g of each sample and 20 mL of distilled water were properly mixed before being put into centrifugal tubes. The mixture was centrifuged for 10 min at 700 × g at 4°C (Eppendorf 5702R, Merck, Darmstadt, Germany). The recovered supernatant was then dried at room temperature ± 24°C to achieve consistent mass. The dry material will be treated as a solid mass in the supernatant.

##### 2.2.3.10 Dispersibility

Dispersibility was estimated using the reported method by Ohizua et al. (32) of the flour samples. About 10 g of each sample was added to the pre-dried 100 mL cylinder and was filled with distilled water until 100 mL mark. The obtained mixture was stirred and left to stand for 3 h and the dispersibility of the flours was expressed as percentage.

##### 2.2.3.11 Colour profile

The colour characteristics (L*, a*, b*, and ∆E) of the flours were measured using a dual system consisting of a measuring head and a data processor (CR-410 and DP-400 Ver. 1.20, Konica Minolta, Inc., Tokyo, Japan). Prior to analysing the flour samples, the device was calibrated using a white tile (refractive index was mentioned). The flour samples were analysed for their lightness (L*), redness/greenness (a*), yellowness/blueness (b*), and overall colour differences (∆E*) (21). In addition, the browning index was determined using Equations 3 and 4 below:


(3) $$\text{BI}=\frac{\left[100\left(y-0.31\right)\right]}{0.17}$$

(4) $$y=\frac{\left(a*+1.75L*\right)}{\left(5.645L*+a*-3.012b*\right)}$$

where BI = browning index, L = lightness, a = redness, b = yellowness.

#### 2.2.4 Statistical analysis

All generated data for each analysis was performed on one-way analysis of variance software (IBM SPSS, ver. 26.0, New York, United States) at *p* < 0.05. The results were shown as mean ± standard deviation (SD). The unsupervised principal component analysis (PCA; SIMCA 18, Umetrics, Umea, Sweden) was used to visualise grouping the patterns and also to identify the outliers in data sets.

## 3 Results and discussion

### 3.1 Effect of processing (fermentation, malting and ultrasonication) on proximate composition

The proximate composition of food is usually used for the estimation of the quantitative analysis of food substance which includes ash, moisture, fibre, fat, protein and total carbohydrates (34). The macronutrients of the samples are presented in Table 1, and it can be observed that malted sorghum flour (MSF) had the highest moisture content while ultrasonicated sorghum flour (USF) had the lowest moisture content. The fermented sorghum flour (FSF) significantly decreased as compared to raw sorghum flour (RSF). Raw mopane worm (RMW) reported the highest moisture content as compared to ultrasonicated mopane worm (UMW) which had the lowest moisture content. Ultrasonicated *M. oleifera* (UMO) reported the highest moisture content while raw *M. oleifera* (RMO) had the lowest moisture content. Mopane worm flour was observed to have more ash content compared to all the other samples that were investigated in the current study. These findings are consistent with what is reported by Kewuyemi & Adebo (30), where no significant difference between the processes in all samples was observed.

**Table 1:** Proximate composition and energy content of raw, fermented, malted and ultrasonicated sorghum, mopane worm and moringa flours.

Sample	Ash (%)	Moisture (%)	Protein (%)	Fibre (%)	Fat (%)	Carbohydrate (%)	Energy (kcal/g)
RSF	1.53 ± 0.00^a^	9.93 ± 0.02^h^	12.77 ± 0.03^ab^	3.73 ± 0.01^a^	3.35 ± 0.17^b^	68.69 ± 0.16^f^	363.48 ± 0.75^c^
MSF	1.49 ± 0.00^a^	10.39 ± 0.18^i^	12.46 ± 0.05^a^	3.69 ± 0.01^a^	3.35 ± 0.12^b^	68.62 ± 0.01^f^	361.87 ± 1.27^c^
FSF	1.77 ± 0.01^b^	3.24 ± 0.05^b^	13.02 ± 0.05^b^	4.23 ± 0.00^b^	3.35 ± 0.05^b^	74.39 ± 0.05^g^	388.27 ± 0.39^de^
USF	1.83 ± 0.01^c^	2.80 ± 0.06^a^	13.38 ± 0.13^c^	4.53 ± 0.07^c^	2.82 ± 0.03^a^	74.64 ± 0.10^g^	386.50 ± 0.25^d^
RMW	10.34 ± 0.02^f^	7.29 ± 0.04^e^	51.59 ± 0.35^f^	9.19 ± 0.02^d^	15.57 ± 0.20^e^	6.02 ± 0.26^c^	388.98 ± 0.86^e^
FMW	11.15 ± 0.02^g^	5.91 ± 0.03^d^	52.39 ± 0.25^g^	11.28 ± 0.02^f^	15.60 ± 0.34^e^	3.66 ± 0.44^b^	387.21 ± 1.95^de^
UMW	11.10 ± 0.01^g^	5.14 ± 0.12^c^	56.24 ± 0.18^h^	11.74 ± 0.05^g^	15.20 ± 0.06^e^	0.58 ± 0.35^a^	387.52 ± 0.21^de^
RMO	7.88 ± 0.01^e^	8.63 ± 0.09^f^	30.68 ± 0.16^e^	10.00 ± 0.02^e^	4.13 ± 0.48^c^	38.68 ± 0.27^e^	334.63 ± 2.63^b^
UMO	7.46 ± 0.08^d^	9.13 ± 0.00^g^	29.32 ± 0.32^d^	13.86 ± 0.01^h^	4.64 ± 0.19^d^	35.59 ± 0.21^d^	329.13 ± 0.83^a^

RSF, raw sorghum flour; FSF, fermented sorghum flour; MSF, malted sorghum flour; USF, ultrasonicated sorghum flour; RMW, raw mopane worm; FMW, fermented mopane worm; UMW, ultrasonicated mopane worm; RMO, raw *Moringa oleifera*; UMO, ultrasonicated *Moringa oleifera*. Values in brackets are standard deviations of the respective means with different superscripts which are significantly different (*p* ≤ 0.05) per column.

The USF, UMW and UMO showed significant increases in fibre content as compared to other processed samples and raw samples. MSF was reported to have decreased with no significant difference with RSF. As expected, RMW had the highest protein content among all the samples reported, with UMW (56.24%) being the highest. This can be due to ultrasonication creating mechanical vibrations when applied to food, disrupting the structure of the food matrix. This disruption causes the release of proteins that are attached to food particles or cellular structures. As a result, more proteins become accessible in the liquid phase, hence boosting the total protein content (35). The same trend is also observed in sorghum but not in MO and this could be due to the disruption of sensitive cells in plants. Proteins contain complex molecules that are susceptible to mechanical and chemical stress therefore denaturing or degrading the proteins, resulting in protein loss rather than an increase (36).

Among all the samples studied, RSF contained less amount of fat as expected and MW had the highest fat content. This is due to mopane worms feeding mainly on mopane tree leaves that are high in lipids, namely unsaturated fatty acids like oleic and linoleic acid. As a result, the caterpillars absorb lipids from their food, which contributes to their high-fat content (37). Mopane worms usually store their energy as fats rather than carbohydrates hence the carbohydrates for RMW it ranged from 0.58–6.02% which was the lowest in all samples. Fat offers a more concentrated and efficient energy reserve than carbohydrates, making it a favoured storage type, especially in insects that undergo substantial growth and developmental changes. Grains have been reported to contain more carbohydrates than other food. The carbohydrates recorded for RSF ranged from 68.62–74.64% with USF being the highest and no significant difference was observed between FSF and USF as well as in energy levels. The MO samples showed the lowest energy levels 329.13–334.63 kcal.

### 3.2 Effect of processing (fermentation, malting and ultrasonication) on biochemical properties

The fermentation process significantly (*p* ≤ 0.05) decreased pH in FSF (4.32) and FMW (4.76) as observed in Table 2 compared to the control, malting and ultrasonication. With MO, the UMO had the lowest pH (5.57) compared to the RMO. As expected, when the pH decreased, the TTA correspondingly increased as well. The highest TTA observed in Table 1 for RSF was the FSF with the lowest pH while the lowest recorded was seen in RSF (0.07% lactic acid) which had the highest pH (6.23). The same trend was also observed in RMW as well as in RMO. According to Van Kerrebroeck et al. (38), the biomodified flour’s pH and acidity are influenced by both endogenous (enzymes and bacteria) and external variables (dough yield, temperature, and time). Similar trends were also observed in the study by Kewuyemi et al. (21).

**Table 2:** pH, TTA, TPC, TFC and antioxidant activities of raw, fermented, malted and ultrasonicated sorghum, mopane worm and moringa flours.

Sample	pH	TTA (% lactic acid)	TPC (mg GAE/g)	TFC (mg QE/g)	ABTS (mM TE/g)	FRAP (mM TE/g)
RSF	6.23 ± 0.01^i^	0.07 ± 0.06^a^	0.79 ± 0.01^a^	1.27 ± 0.03^a^	11.68 ± 0.01^b^	5.73 ± 0.02^g^
MSF	5.29 ± 0.03^c^	0.18 ± 0.06^c^	3.23 ± 0.02^e^	3.05 ± 0.03^d^	28.38 ± 0.01^d^	1.19 ± 0.02^b^
FSF	4.32 ± 0.01^a^	0.24 ± 0.11^d^	1.10 ± 0.06^b^	1.70 ± 0.03^b^	18.71 ± 0.02^c^	5.43 ± 0.02^f^
USF	6.14 ± 0.01^h^	0.17 ± 0.06^b^	1.63 ± 0.03^c^	2.17 ± 0.02^c^	10.02 ± 0.02^a^	3.79 ± 0.02^e^
RMW	5.76 ± 0.01^f^	0.49 ± 0.17^h^	3.63 ± 0.02^f^	6.98 ± 0.02^e^	31.64 ± 0.01^g^	3.10 ± 0.03^d^
FMW	4.76 ± 0.01^b^	0.69 ± 0.06^i^	6.18 ± 0.03^g^	8.84 ± 0.01^g^	32.49 ± 0.01^i^	0.41 ± 0.03^a^
UMW	5.86 ± 0.01^g^	0.42 ± 0.06^g^	3.09 ± 0.03^d^	8.57 ± 0.03^f^	32.27 ± 0.02^h^	2.61 ± 0.03^c^
RMO	5.71 ± 0.01^e^	0.30 ± 0.06^f^	21.47 ± 0.03^i^	74.79 ± 0.01^i^	29.67 ± 0.03^e^	55.30 ± 0.03^i^
UMO	5.57 ± 0.01^d^	0.29 ± 0^e^	11.60 ± 0.03^h^	74.02 ± 0.02^h^	30.87 ± 0^f^	44.12 ± 0.02^h^

TTA, titratable acidity; TPC, total phenolic content; TFC, total flavonoid content; ABTS, 2,2-Azinobis-(3-ethylbenzthiazolin-6-sulfonic acid); FRAP, Ferric ion reducing antioxidant power; RSF, raw sorghum flour; FSF, fermented sorghum flour; MSF, malted sorghum flour; USF, ultrasonicated sorghum flour; RMW, raw mopane worm; FMW, fermented mopane worm; UMW, ultrasonicated mopane worm; RMO, raw *Moringa oleifera*; UMO, ultrasonicated *Moringa oleifera*. Values in brackets are standard deviations of the respective means with different superscripts which are significantly different (*p* ≤ 0.05) per column.

The ultrasonicated samples had the highest pH as compared to fermented samples. This is because, during fermentation, bacteria metabolise glucose and other chemicals in the food substrate. As a result of this metabolic process, they create organic acids such as lactic acid, acetic acid, and citric acid. These organic acids are acidic and help to reduce the pH of fermented foods. This reduction in pH limits the growth of spoilage microbes and diseases causing pathogens thereby preserving the quality of food (21). Ultrasonication, on the other hand, is a mechanical process that disrupts cells, emulsifies chemicals, and aids in extraction. While ultrasonication can change the physical features of food, such as texture or particle size, it has no direct impact on the chemical content or pH of the meal. As a result, ultrasonicated meals seldom undergo pH changes like fermented foods (39).

Food processing has been reported to increase the nutritional and health-promoting properties of food (40). Table 2 shows the TPC, TFC and antioxidant activities of the flour samples. As observed, malting had the highest TPC (3.23 mg GAE/g), TFC (3.05 mg QE/g) and ABTS (28.38 mM TE/g) among all the investigated processes in sorghum and reported the lowest in FRAP (1.19 mM TE/g). These alterations may be due to the increased release or enzymatic production (i.e., proteolysis) of soluble bioactive molecules with a higher potential to scavenge free radicals (41). The same trend was also observed in RMW, fermented RMW had the highest TPC (6.18 mg GAE/g), TFC (8.84 mg QE/g) and ABTS (32.49 mM TE/g) but reported the lowest in FRAP (0.41 mM TE/g). As expected, MO had the highest TPC, TFC and FRAP among all the other samples. However, the composition of the insoluble proteolytic products and non-phenol components produced in the samples might impact the considerable reduction (*p* < 0.05) of FRAP (0.41 mM TE/g) in FMW (41).

### 3.3 Effect of processing (fermentation, malting and ultrasonication) on physical properties and colour

The effect of processing of SF, MW and MO on physical properties and colour are presented in Tables 3, 4. The significance of fermented, malted and ultrasonicated flours for post-processing mostly depends on their excellent physical properties (42). The FSF (2.50%) showed a significant WHC increase (*p* ≤ 0.05) as well as in OHC (2.53) while UMW showed a significantly higher WHC and OHC (3.11 and 3.20 g/g, respectively). Among all the investigated samples, RMO had the highest WHC (3.76 g/g) but showed the lowest OHC (1.26 g/g). This is because many plant tissue components such as polysaccharides, proteins, and pectin, are hydrophilic, which means they prefer water. These chemicals can absorb and hold water within the plant’s cells and tissues (43). The FSF and USF had a significantly higher (*p* ≤ 0.05) dispersibility (87.43 and 87.87%) as compared to other samples with MO being the lowest. According to Eke-Ejiofor (44), a high dispersibility can enhance the better reconstruction of starch in water to give a fine and constituent paste.

**Table 3:** Physical properties of raw, fermented, malted and ultrasonicated sorghum, mopane worm and moringa flours.

Sample	WHC (g/g)	OHC (g/g)	Dispersibility (%)
RSF	1.94 ± 0.70^a^	2.25 ± 0.01^bc^	86.83 ± 0.29^f^
MSF	2.12 ± 0.36^ab^	2.11 ± 0.35^b^	86.00 ± 0^e^
FSF	2.50 ± 0.32^b^	2.53 ± 0.32^cd^	87.43 ± 0.12^g^
USF	2.29 ± 0.06^ab^	2.44 ± 0.05^bcd^	87.87 ± 0.23^h^
RMW	2.58 ± 0.01^bc^	2.62 ± 0.01^d^	80.33 ± 0.29^c^
FMW	2.53 ± 0.01^b^	2.54 ± 0.01^cd^	81.00 ± 0^d^
UMW	3.11 ± 0.28^c^	3.20 ± 0.32^e^	85.80 ± 0.35^e^
RMO	3.76 ± 0.07^d^	1.21 ± 0^a^	70.73 ± 0.23^a^
UMO	2.66 ± 0.07^bc^	1.26 ± 0.01^a^	73.23 ± 0.40^b^

WHC, water holding capacity; OHC, oil holding capacity; RS, raw sorghum; FS, fermented sorghum; MS, malted sorghum; US, ultrasonicated sorghum; RMW, raw mopane worm; FMW, fermented mopane worm; UMW, ultrasonicated mopane worm; RMO, raw *Moringa oleifera*; UMO, ultrasonicated *Moringa oleifera*. Values in brackets are standard deviations of the respective means with different superscripts which are significantly different (*p* ≤ 0.05) per column.

**Table 4:** Colour attributes of raw, fermented, malted and ultrasonicated sorghum, mopane worm and moringa flours.

Sample	L*	a*	b*	∆E*	BI
RSF	77.25 ± 0.09^g^	3.19 ± 0.01^b^	12.65 ± 0.04^c^	50.71 ± 0.08^g^	20.55 ± 0.10^b^
MSF	77.99 ± 0.03^h^	4.43 ± 0.02^e^	12.85 ± 0.11^d^	51.56 ± 0.06^h^	21.81 ± 0.18^c^
FSF	79.09 ± 0.02^i^	3.64 ± 0.02^c^	12.47 ± 0.04^b^	52.46 ± 0.03^i^	20.18 ± 0.07^a^
USF	74.34 ± 0.12^f^	3.62 ± 0.02^c^	11.58 ± 0.07^a^	47.64 ± 0.13^f^	20.17 ± 0.09^a^
RMW	58.43 ± 0.10^d^	2.69 ± 0.01^a^	24.29 ± 0.05^i^	39.22 ± 0.05^e^	55.65 ± 0.29^e^
FMW	58.55 ± 0.05^e^	2.66 ± 0.09^a^	23.85 ± 0.01^h^	39.02 ± 0.01^d^	54.25 ± 0.20^d^
UMW	42.57 ± 0.01^b^	3.58 ± 0.02^c^	17.89 ± 0.06^e^	23.71 ± 0.05^a^	59.36 ± 0.28^f^
RMO	46.36 ± 0.01^c^	10.51 ± 0.01^f^	23.33 ± 0.02^g^	32.12 ± 0.05^c^	84.60 ± 0.13^h^
UMO	38.99 ± 0.03^a^	3.82 ± 0.03^d^	20.23 ± 0.05^f^	24.06 ± 0.05^b^	77.71 ± 0.10^g^

L*, lightness; a*, redness; b*, yellowness; ∆E, total colour difference; BI, browning index; RSF, raw sorghum flour; FSF, fermented sorghum flour; MSF, malted sorghum flour; USF, ultrasonicated sorghum flour; RMW, raw mopane worm; FMW, fermented mopane worm; UMW, ultrasonicated mopane worm; RMO, raw *Moringa oleifera*; UMO, ultrasonicated *Moringa oleifera*. Values in brackets are standard deviations of the respective means with different superscripts which are significantly different (*p* ≤ 0.05) per column.

Colour profiling is a key indicator of food quality and customer preferences. Table 4 represents the colour profiling of the investigated samples. The study found that RMO and UMO samples exhibited significantly higher (*p* ≤ 0.05) redness (10.51–3.82) and browning index (77.71–84.60), but lower total colour difference (24.06–32.12). In contrast, raw, fermented, and malted red sorghum flour (RSF) samples showed significant increases in lightness (74.34–79.09), while RMW as well as FMW had a higher yellowness (24.29 and 23.85 respectively). The differences are likely influenced by the seed coat colour of sorghum grains and the natural colour of mopane worms. The UMW had a significant decrease in lightness (42.57) but had an increased redness (3.58).

The maximum redness of the UMW might have led to its browning index of 59.36. The higher browning index indicates the extent of browning impacted by ultrasonication. In other words, the RMW ultrasonication improved its redness and browning index and a lesser intensity of yellowness and reduced lightness. Plants exhibit a higher amount of redness in colour profile than other types of food due to the presence of pigments including anthocyanins, lycopene, and betacyanins, as well as environmental and genetic influences (45). This might have resulted in the MO’s highest redness and browning index, and consequently having the lowest lightness. The colour profile results also showed that malting increases the lightness of MSF (77.09) compared to USF (74.34). The observed differences are partly related to the earlier reported low USF’s TPC (1.63 mg GAE/g; Table 1), suggesting that the leaching of polyphenol constituents during malting steps improved the lightness of MSF.

### 3.4 Principal component analysis

**Figure 1:**
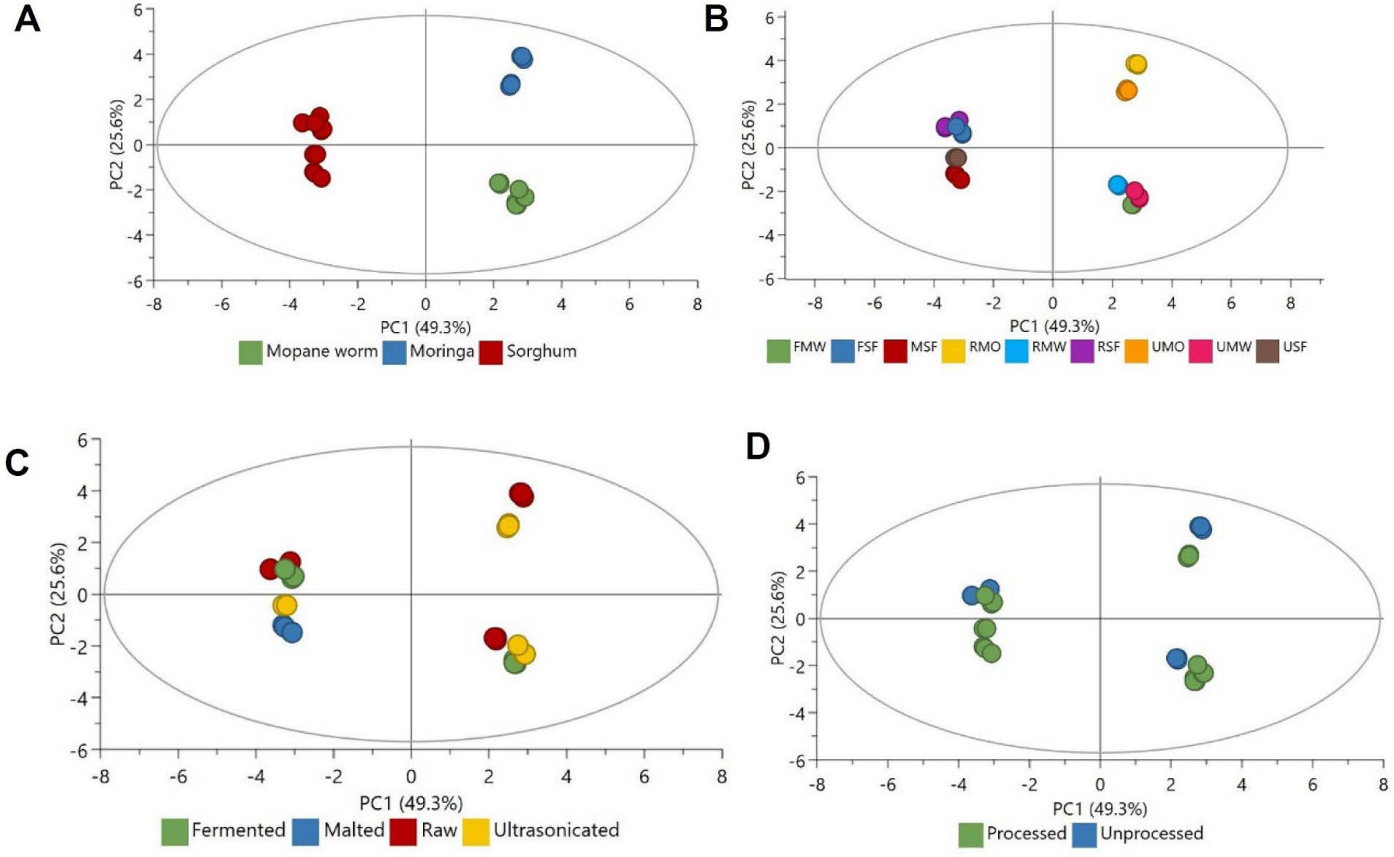
PCA score plots showing distribution and separation. **(A)** Relationship of mopane worm, moringa and sorghum, **(B)** relationship of the differently processed samples, **(C)** relationship of raw, fermented, malted and ultrasonicated, **(D)** relationship of processed and unprocessed.

An unsupervised modelling technique (PCA) was used to understand and highlight the groupings of the samples in this study, based on the investigated parameters. This allowed for an exploratory statistical analyses and descriptive assessment of the data showing trends and relationships as depicted in Figures 1A–D. The first two principal components (PCs), PC1 and PC2 explained 49.3 and 25.6% of the variation (total of 74.9%). Figure 1A shows a distinct separation of sorghum (to the left of the plot), moringa (to the top right) and mopane worm (to the bottom left). This is in alignment to the inherent composition and characteristics of these food sources, reflected in the obtained data on Tables 1–4.

The groupings also reflect the unique groupings of the substrates investigated in the current study: sorghum (being a cereal), moringa (being a medicinal plant) and mopane (being an edible insect). While still retaining the clusters, Figure 1B shows that the processes (fermentation, malting and ultrasonication) affected the properties of the samples. As observed in Figure 1D, PC1 separated more of the processed samples to the left while the unprocessed samples were shifted to the right. Most the processed samples were clustered in the lower part of the PC1 as compared to the unprocessed. The identified separation and clusters could be due to biochemical activities that occurred during the fermentation, malting, and ultrasonication processes.

## 4 Conclusion

The present study explored traditional (fermentation and malting) and novel (ultrasonication) processes to improve the quality properties of sorghum, mopane worm and *M. oleifera* flours. The processes were found to have caused variations in the biochemical, nutritional and functional properties of the investigated flours. As expected, fermentation of flours led to low acidity levels and higher TTA than other processes. Higher TPC and TFC were observed in malting corresponding in higher antioxidant (ABTS) for sorghum while for mopane worms it was observed in fermentation then raw moringa had the highest TPC and TFC. The ultrasonication of RSF and RMW resulted in higher contents of ash, protein and fibre. The biomodification by fermentation in sorghum led to higher WHC and OHC and increased dispersibility in ultrasonication. In mopane worms, ultrasonication led to higher WHC, OHC and dispersibility. Lightness was greater in the fermented samples of RSF and RMW while raw MO had the greatest lightness than UMO. Among all samples, MO had the most redness and browning index. Ultrasonication has proven to be more effective in improving the nutritional value in the samples, followed by fermentation. The blend of bioprocessed flours with various nutritional and health properties can help address the challenges of malnutrition by developing finished products from these flours. It can be recommended to further investigate the technological features of the processed flours to help compose the formulations.

## Data Availability

The raw data supporting the conclusions of this article will be made available by the authors, without undue reservation.
